# Thematic series CAPMH “Forensic Child and Adolescent Psychiatry and Mental Health 2017”

**DOI:** 10.1186/s13034-018-0213-8

**Published:** 2018-01-15

**Authors:** Cyril Boonmann, Jörg M. Fegert, Klaus Schmeck

**Affiliations:** 1 Child and Adolescent Psychiatric Research Department, Psychiatric University Hospitals Basel, Basel, Switzerland; 2 Department of Forensic Child and Adolescent Psychiatry, Psychiatric University Hospitals Basel, Basel, Switzerland; 3 Child and Adolescent Psychiatric Hospital, University of Ulm, Ulm, Germany

## Editorial

Since the end of the last century, there has been an increasing focus on tailored assessment and treatment of juvenile offenders with psychiatric and psychosocial problems. These include, among others, forensic psychiatric evaluations in which the development of the juvenile is taken into account, psychotherapeutic treatment in forensic psychiatric centres for young offenders, as well as in an ambulatory setting, psychiatric support in juvenile prisons. This increased attention is also reflected in a steady increase in the number of scientific publications in the field of forensic child and adolescent psychiatry and psychology since the 1990s.

The founding of the European Association for Forensic Child and Adolescent Psychiatry, Psychology and other involved professions (EFCAP) (http://www.efcap.org/) in 1997 is in line with this development of the field. The aims of EFCAP are: (1) to improve joint international research, training and education, (2) to exchange research data and practical experiences as well as to innovate research and treatment methods, (3) to contribute to national and European policy and (4) to raise awareness of the need for changes in the criminal and civil justice system as well as to provide the best possible care for the juveniles within these systems.

Based on these aforementioned objectives, an annual thematic series has been established in collaboration with Child and Adolescent Psychiatry and Mental Health (CAPMH), of which previous editions have been published in 2011 [1] and 2016 [2]. EFCAP is proud to present the thematic series of “Forensic Child and Adolescent Psychiatry and Mental Health” of this year. The current edition consists of six papers with a broad range of forensic child and adolescent psychiatry/psychology issues.

In the first paper of this thematic series, Leenarts et al. [3] studied the relationship between psychopathic traits and delinquency in boys and girls from the general population and from a high-risk sample for juvenile delinquency. One of their main goals was to examine whether the moderating effect of gender on the relationship between psychopathic traits and self-reported delinquency was comparable in both samples. They conducted their research in a sample of more than 1200 adolescents of the Germans speaking part of Switzerland. Psychopathic traits were assessed by means of the Youth Psychopathic traits Inventory (YPI), delinquency by means of a self-reported delinquency instrument.

The second paper, of Janssen-de Ruijter et al. [4], focused on the co-occurrence of risk factors in subgroups of juveniles in residential care (i.e. hospital for youth forensic psychiatry and orthopsychiatry) in the Netherlands in order to better understand the concept of disruptive behavior. Based on the Structured Assessment of Violence Risk in Youth (SAVRY) and the Juvenile Forensic Profile (JFP) they operationalized various risk factors within the domains: individual, family, peer and school. They were able to distinguish between four classes with different patterns of co-occurring risk factors.

On the same page, Hillege et al. [5] examined the heterogeneity of juvenile offenders in juvenile justice institutions in the Netherlands. This, as the variety of their risk factors and personal characteristics complicates treatment planning. In a sample of more that 2000 juvenile offenders, they identified seven subgroups that were not only of scientific relevance but also of clinic relevance.

Next, Van Duin et al. [6] explored the transition phase from adolescence to emerging adulthood retrospectively in a sample of multi-problem young adults. As many of these young adults have been exposed to childhood maltreatment or displayed adolescent offending behaviour, they might have come into contact with the Child Protection Service (CPS). The aim of this fourth contribution was to study the CPS data of multi-problem young adults and to examine its relationship with current mental health problems and offending behaviour. Based on latent class analysis, the authors were able to identify four classes.

In the fifth paper, Barendregt et al. [7] examined two assumptions of the Good Lives Model: (1) whether subjective Quality of Life (QoL) is related to offending behavior and psychosocial problems, and (2) whether adolescents with adequate coping skills are less likely to show these behaviors and problems. They based their results on four-wave longitudinal study with a sample of 95 adolescents with severe psychiatric problems. QoL was assessed with the Lancashire Quality of Life Profile, coping skills with the Utrecht Coping List for Adolescents.

Finally, Simons et al. [8] present a newly developed family-centred care program for adolescents in short-term stay groups in juvenile justice institutions. This program distinguishes four categories of parental participation: (1) informing parents, (2) parents meeting their child, (3) parents meeting staff, and (4) involvement in treating program.

In the current issue, five of the six papers are from the Netherlands. This reflects the high standard of adolescent forensic psychiatry in clinical care as well as the prominent role of research in the Netherlands. A good example of an ongoing project in the Netherlands in which science and practice work together to improve the field of juvenile forensic care is the Academic Workplace for Forensic Care for Youth (see for example: [9, 10]). This Academic Workplace was a collaborative venture between two juvenile justice institutions, two mental health care institutions, two university departments of child and adolescent psychiatry and two universities of applied sciences. The project continues as an Academic Workplace for Risk Youths in which the municipalities will also take an important role (for more information: http://awrj.nl). We hope that these enormous efforts that have been undertaken in the last decade in the Netherlands will stimulate researchers and clinicians from other European countries to establish similar “academic workplaces” to develop the field of adolescent forensic care.

To target the special needs of children and adolescents with both mental health problems and delinquent behaviour we need a comprehensive understanding of risk factors and protective factors over the developmental course of these young people. The gold standard to detect these factors are longitudinal research designs which should not only focus on reoffending as outcome criterion, but also on both mental and physical health, as well as on psychosocial functioning and quality of life. The Dunedin Study [11] or the Great Smoky Mountain Study [12] are fascinating landmark studies with outstanding contributions to our understanding of the developmental course of disorders. On a smaller scale, there are European longitudinal studies like the Basel longitudinal study on mental health problems and offending behaviour in young people from residential care settings (Project Youth Welfare Pathways: Learning from Experience, in German: Jugendhilfeverläufe: Aus Erfahrung Lernen [JAEL], which is a follow up of the Project Clarification and achievement of objectives in stationary measures, in German: Abklärung und Zielerreichung in stationären Massnahmen [MAZ.]) [13] (for more information, see: http://www.jael-portal.org) or the European FemNat-study [14] that is focused on girls with externalizing and delinquent behaviour (for more information, see: http://www.femnat-cd.eu). The 2018 thematic series will have a focus on longitudinal research and we invite researchers in the field of adolescent forensic psychiatry and psychology to contribute to next year’s special issue.

Finally, the sixth EFCAP Congress in Venice (20–22 June 2018) will give the opportunity to get into a close exchange to start or to broaden scientific collaborations and to share new approaches in assessment and clinical care of young offenders suffering from mental problems. The EFCAP board cordially invites you to attend this biannual conference. (For more information: http://www.efcap2018.com).
